# Hinst: Human‐Like Interactive Instinct Enables Robots to Robustly Accomplish Universal Tasks

**DOI:** 10.1002/advs.202509483

**Published:** 2025-07-28

**Authors:** Zijian Liao, Qian Mao, Yichen Qin, Jinfeng Yuan, Rong Zhu

**Affiliations:** ^1^ Department of Precision Instrument State Key Laboratory of Precision Measurement Technology and Instruments Tsinghua University Beijing 100084 China

**Keywords:** anthropomorphic architecture, human‐like instinct, intelligent robot, robust manipulation, universal tasks accomplishment

## Abstract

Robots have enormous potential to assist humans in daily life. However, current robot development and popularization are impeded by its deficient functionality, lengthy task deployment, and vast experimental training. Poor flexibility, poor practicality, and poor universality in task accomplishment hinder robot applications. Here, a human‐like interactive instinct (Hinst) and a Hinst robotic architecture are proposed to enable robots to flexibly and robustly accomplish universal tasks in the real world. The Hinst architecture incorporates multimodal senses, logical decision‐making, and initiative task‐execution, casting robots with human‐like instinctive reaction and intelligence expansion. The robot is empowered with instinctive interaction ability relying on its inherent touch‐sense‐feedback, knowledge‐driven cognition, and adaptive‐control, while its high‐level intelligence of task‐planning and action‐skills are nurtured from acquired knowledge‐learning or human‐teaching. The Hinst greatly enhances the efficiency and the success rate of robotic manipulation, skill learning, and task execution. This anthropomorphic architecture offers a generalizable and universal pathway for general‐purpose robots (especially humanoid robots) to robustly accomplish complex housework and industrial skilled work.

## Graphical abstract

Aiming at current robot limitations of poor flexibility, poor practicality, and poor universality in task accomplishment, the authors report a human‐like interactive instinct (Hinst) and an anthropomorphic robotic system architecture. The Hinst greatly enhances the efficiency and the success rate of robotic manipulation, skill learning, and task execution, offering a generalizable and promising pathway for general‐purpose robots.

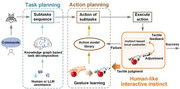

## 1 Introduction

**Figure 1:**
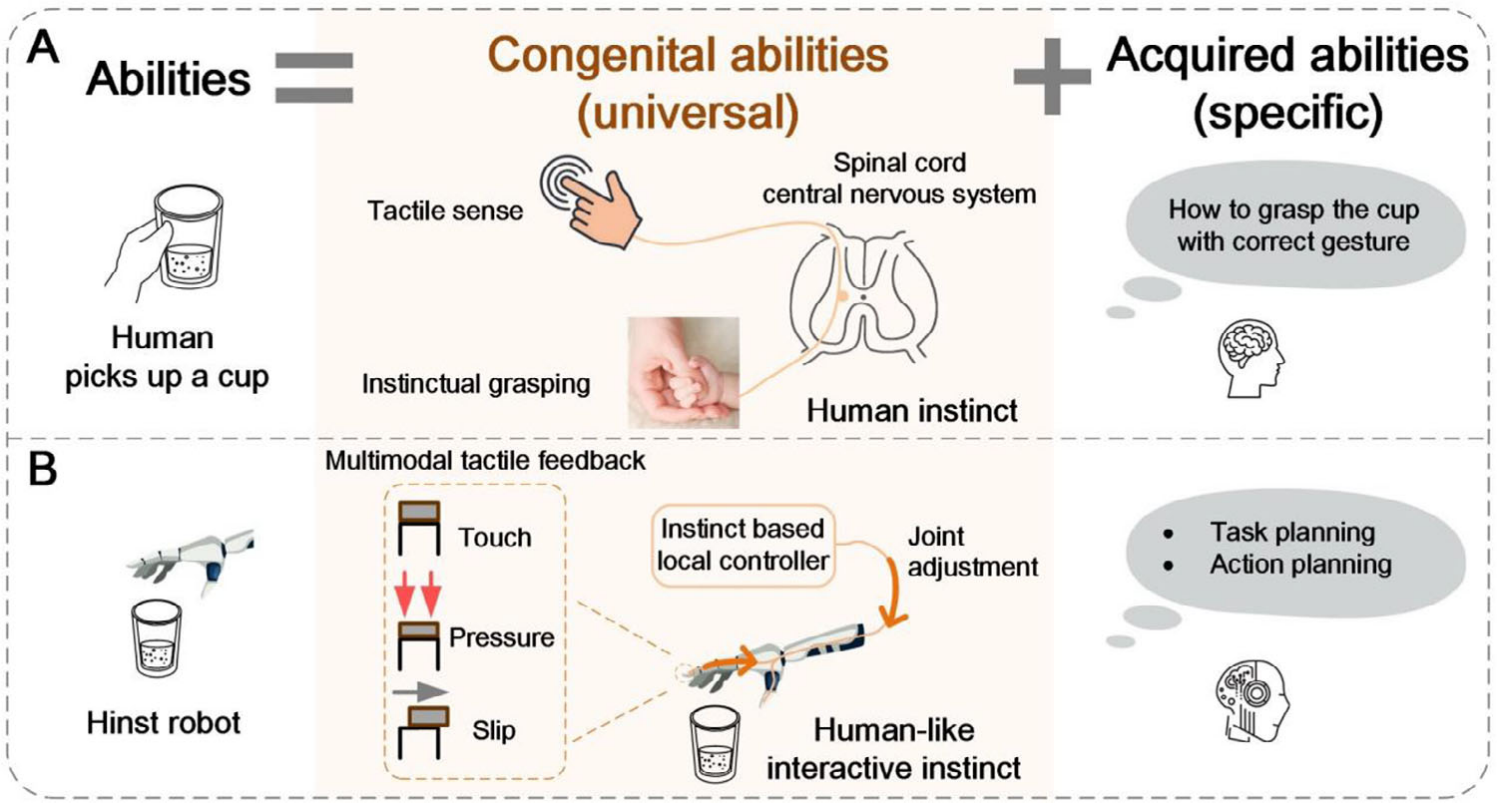
Both humans and robots necessitate interactive instinct. A) Human capabilities are divided into two categories: congenital abilities and acquired abilities, the combination of congenital and acquired abilities enables humans to accomplish complex tasks. B) Our proposed Hinst robot possesses both congenital abilities (tactile sense, instinctive touch‐sense‐feedback) and acquired abilities (task planning, action planning) to accomplish versatile tasks. The robots are equipped with multimodal tactile sensors capable of perceiving contact, pressure, and slip, ensures multimodal tactile feedback control.

Human capabilities contain congenital abilities and acquired abilities (**Figure** **1**A). Humans are innately capable of grasping objects via touch sense. Even newborn infants possess an inborn ability to grasp. Humans rapidly adjust the hand without conscious thought when an object slips from their grasps. These instinctual behaviors, based on fast touch‐sense feedback, are spontaneous without high‐level decision‐making of brain. Being fundamental to environmental interaction, the instinct universally enables individuals adapting to various interactive scenarios swiftly, accurately, and robustly. Building upon it, humans further acquire advanced skills for accomplishing complex tasks. Take picking up a cup as an example (Figure 1A), on one hand, the acquired knowledge enables humans to know how to grasp a paper cup with an appropriate gesture. On the other hand, the innate touch‐sense‐feedback (i.e., interactive instinct) allows humans to pick up the cup with appropriate force, avoiding unnecessary excessive force to crush the cup. If the cup slips during lifting process, humans can rapidly adjust the hand to achieve stable grasp. The combination of instinct and acquired abilities enables humans to universally handle various complex tasks, with neither being dispensable.

Despite the rapid advancements in robot technology, accomplishing complex tasks in the real world remains a significant challenge for robots. Existing studies mainly focus on developing acquired abilities of robots in manipulation learning and task planning. While instinctive interaction ability of robots has been overlooked.

Imitation learning and reinforcement learning are methods employed for robots to acquire manipulation skills.^[^ ^1^, ^2^, ^3^, ^4^, ^5^, ^6^ ^]^ However, these methods face the challenge of poor generalizability, especially for unstructured real‐world environments. Task scenario requires cumbersome data collection and computationally intensive model learning, making it difficult to apply robotic skills across multiple scenarios.

Many researches rely on large language models (LLMs) to deploy tasks.^[^ ^7^, ^8^, ^9^, ^10^, ^11^, ^12^, ^13^ ^]^ Owing to the great potential of LLMs for zero‐shot deployment in open‐world scenarios, they have been widely applied to robotic task planning. LLMs excel at task reasoning and planning, capable of generating executable code or action primitives. Vision language models (VLMs) are also utilized in robots to understand the surrounding or learn skills from human demonstrations.^[^ ^11^, ^14^, ^15^, ^16^ ^]^ Although these studies have achieved remarkable progresses, they still neglect the interactive skill in physical manipulation. As a result, many researches showcase handling regular objects without complex manipulation. Simulation is commonly used in validation experiments of robot tasks,^[^ ^2^, ^3^, ^7^, ^16^ ^]^ where the physical interactions between robot and objects are unreasonably simplified, regardless of fragility, slippage, etc. How to transfer the task execution from the virtual world to the real world is a great challenge.

To realize robust manipulation, tactile sense is essential.^[^ ^17^, ^18^, ^19^, ^20^, ^21^, ^22^, ^23^, ^24^, ^25^, ^26^ ^]^ Although force senses have been concerned and involved in robot tasks, due to complex interactive processes during manipulation, effectively cognizing interactive states and executing interaction control are significantly difficult. Many researchers employ data‐driven modeling using deep neural networks.^[^ ^17^, ^19^, ^21^, ^22^ ^]^ However, the data‐driven method struggles to generalize for handling prior‐unseen objects or scenarios in unpredictable environments. The limitations of current robotic task execution lie in: poor universal use, not generally applicable for a variety of objects and environments; poor flexibility, not generally adaptive to varied prior‐unknown task scenarios; poor practicality, not generally effective in the real‐world applications. These problems greatly impede current development of robotic applications.

Here, we propose a new robotic architecture with human‐like interactive instinct (abbreviated as Hinst) for general‐purpose robotic task accomplishment. Endowed with instinctive tactile perception and interaction control, robots can robustly manipulate various objects, like humans. It further incorporates intelligent task planning and action planning to develop its acquired ability, enabling universal uses in robotic tasks. In other words, the Hinst robot combines congenital abilities (multimodal tactile senses, instinctive touch‐sense‐feedback) with acquired abilities (task planning and action planning) to build its integrity functionality. The Hinst architecture empowers the robot an adaptive interactive instinct, incorporating with a task planning to comprehend abstract tasks, and a data‐efficient learning method to acquire new skills. The fingertips of Hinst robot are equipped with multimodal tactile sensors capable of perceiving contact, pressure, and slip, enabling accurately cognizing interactive states (Figure 1B). Moreover, we propose a touch‐sense‐feedback control strategy for the Hinst to autonomously complete robotic manipulation with various objects. The interactive strategy of Hinst is based on a general‐purpose knowledge reasoning, instead of data‐driven method, and thus capably adapts to universal grasping without extensive training. The practicality and generalizability of interactive instinct are experimentally validated, demonstrating a high success rate (97.2%) in manipulating diverse prior‐unseen objects (55 objects). Leveraging the robotic interactive instinct, we further propose a data‐efficient learning method for robots, which enables robots to learn the cross‐scenario manipulation skill from one‐shot human demonstration flexibly. We will showcase how a human teaches the robot to learn the use of tools to robustly manipulate objects, and how the robot autonomously accomplishes a breakfast service for a human. A summary of our work is shown in Movie S1 (Supporting Information).

## 2 Results

### 2.1 Hinst Robotic Architecture with Congenital Abilities and Acquired Abilities

**Figure 2:**
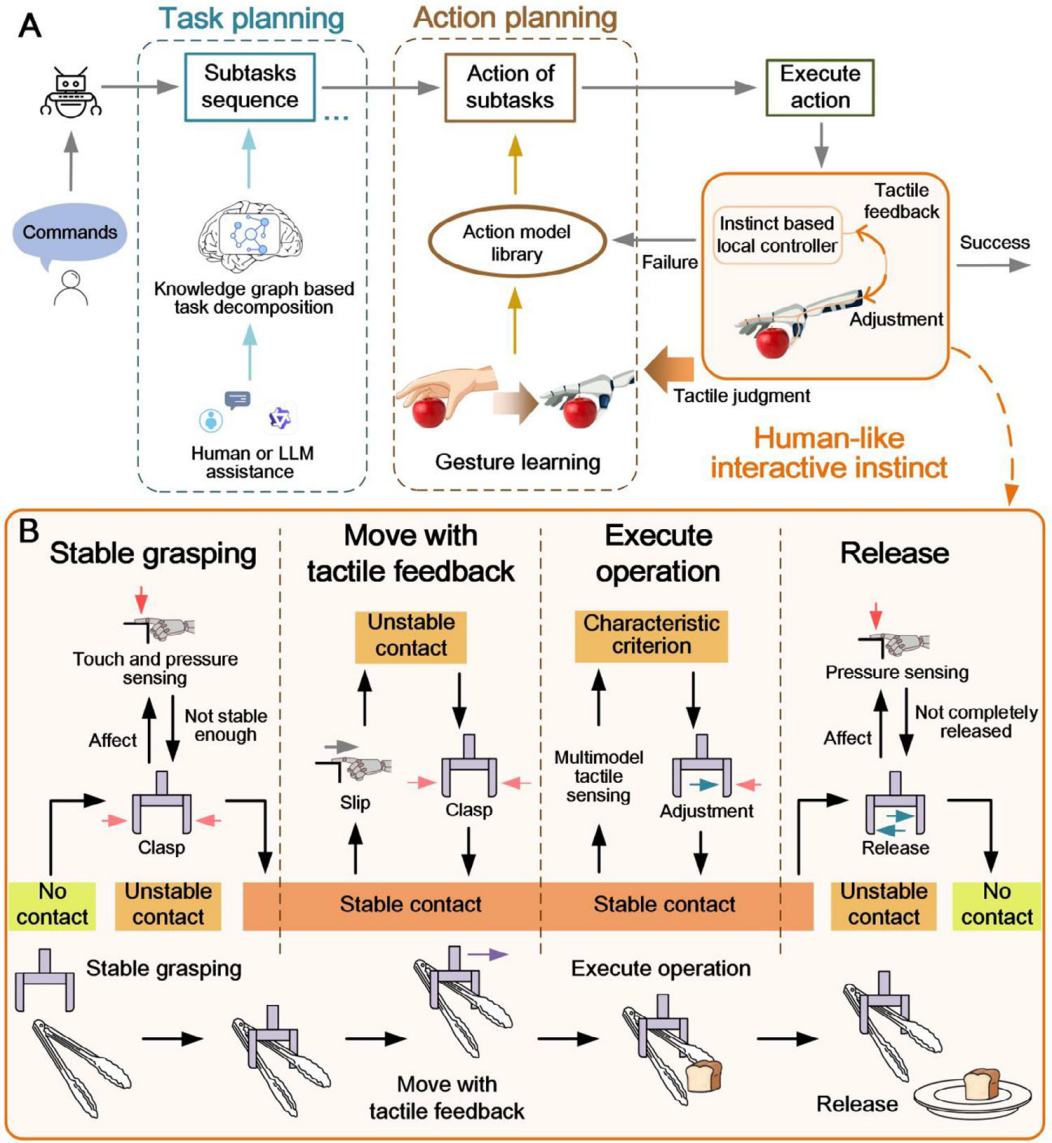
Hinst architecture and the chain of thought in manipulation. A) The Hinst architecture consists of task planning, action planning, and executing action with human‐like interactive instinct. B) The chain of thought for human‐like interactive instinct consists of stable grasping, moving with tactile feedback, executing operation, and release, thereby enabling the robot to accomplish various manipulation tasks with stability and generalization.

The Hinst architecture consists of interactive instinct, task planning, and action planning (**Figure** **2**A), endowing robots with congenital abilities (multimodal tactile senses and instinctive touch‐sense‐feedback control) and acquired abilities (task planning and action planning). The interactive instinct represents an innate capability of the robot to autonomously manipulate objects, while task planning and action planning are the high‐level abilities that the robot acquires through consultation or learning. The innate capability implies a hardcoded behavior and is universal. Once the robot is endowed with the instinct, the instinct applies to all manipulations without subsequent adjustments. In contrast, the acquired abilities can be further optimized in real‐world scenarios. This architecture ensures that the robot can flexibly handle various tasks/actions from knowledge‐driven task planning to Hinst‐based task execution.

As Figure 2A shows, when the Hinst robot receives a task command, it first engages into the task planning. Leveraging a knowledge graph deployed locally on the robot, it decomposes the abstract human commands into a sequence of executable subtasks. Subsequently, the robot enters the action planning and performs the actions of the subtasks, which can be drawn from its locally deployed action model library. Finally, the Hinst robot goes into the execution of actions with its interactive instinct based on its fast tactile feedback to ensure robust manipulation (Details are described in the section 2.2.). The robotic interactive instinct is universally applicable to dexterously manipulate various objects, which allows the success of robotic task execution. Figure S1 (Supporting Information) illustrates the hierarchical structure of the Hinst architecture, from the bottom interactive instinct to the top‐level decision‐making.

In the phase of task planning, the locally deployed task graph facilitates the rapid planning based on the knowledge graphs. When the local task graph fails to meet the planning requirements of the task, the robot may seek the assistance from the cloud LLMs or nearby humans to implement the task planning. Although the LLM method has been widely employed for robot task planning, its task execution efficiency is still unsatisfied. Therefore, we use a locally‐deployed task graph incorporating with human guidance to enhance the robotic efficiency and flexibility. Details are in Experimental Section.

In the phase of action planning, the subtasks decomposed in the task planning are aligned with the actions from the action model library. This library encompasses a range of basic robotic modules, including but not limited to object picking, visual locating, and approaching (Details are available in Experimental Section). When the robot confronts with unprecedented tasks that have not been included in the action model library, the robot may ask for humans to teach. We propose a one‐shot learning method incorporating vision with Hinst. The robot imitates the human's action captured by the vision, meanwhile the robotic interactive instinct assists in accomplishing the tasks. It is worth mentioning that the robot does not need to precisely imitate human's movement, but roughly refers to human's gesture, and the interactive instinct ensures the success of the manipulation tasks (Details are in the section 2.2.). After the success of robotic implementation, the human‐taught gesture data of the manipulation is recorded in the action model library as action primitive for subsequent uses.

In the phase of executing actions, the robot needs to autonomously accomplish a series of manipulation actions. Humans manipulate objects using delicate tactile sense and rapid neural feedback, which executes instinctively. Inspired by human's instinct touch‐sense‐feedback, we propose a human‐like interactive instinct (Hinst) strategy enabling robot to complete object manipulations autonomously. We employ multimodal tactile sensors developed by ourselves,^[^ ^27^, ^28^ ^]^ allowing robot to simultaneously perceive contact pressure and contact interface (contact and slip) during the manipulation. The tactile sensor is composed of two sensing layers: the upper layer (referred as interface sensor) perceives contact interface including touch and slippage, while the lower layer (referred as pressure sensor) perceives contact pressure. The details of the tactile sensor are in the Experimental Section. The tactile sensors are equipped on the fingertips of robot hand. We develop a local controller based on tactile senses, which serves as the robotic neural control center for general‐purpose robotic manipulation, facilitating rapid local closed‐loop feedback control. This interactive capability, akin to human instinct, is an instinct of the robot. The robotic interactive instinct can operate in parallel with the higher‐level decision‐making of robot, and thus ensures the stability, robustness, and practicality of robotic manipulation. Referring to human behavioral habits when solving complex problems through self‐adapting via trial‐and‐error in practice, the Hinst robot also incorporates a trial‐and‐error strategy that if the robot fails in a task, it autonomously adjusts its gesture and retries the task until it is successfully accomplished.

Figure 2B illustrates a chain of thought of the Hinst. The robot perceives the interactive state in real time and autonomously adjusts the grip force when the contact stability is compromised to ensure the stable grasping throughout the whole manipulation process. We categorize the grasping state into: no contact state, unstable contact state, and stable contact state. The unstable contact state typically occurs in the transition between no contact and contact, for example when a slipping occurs. The interactive instinct endows robots stably manipulating objects via the tactile feedback. We phase the thought chain of the interactive instinct into four steps inspired by human's manipulation logic:^[^ ^29^ ^]^ stable grasping, moving with tactile feedback, executing task operation, and release (Figure 2B). Most robotic tasks can be executed via this chain of thought. We develop this robotic instinctive capability with the tactile sensing and proactive control to eliminate unstable states during robotic manipulations, which is embedded in all manipulation tasks to ensure their stability and robustness as illustrated in Figure 2B.

The first step on the chain of thought is stable grasping, where the interactive state between the robotic hand and the object transitions from no contact to stable contact. At the beginning of the grasping, the state is unstable. The Hinst robot perceives the grasping state by the tactile sense and then incrementally increases its grip force until a stable contact is achieved (Details in Experimental Section). It is noted that, considering the object may be fragile, the robot needs to avoid applying excessive force to prevent damage to the object. Therefore, the grip force should be kept as small as possible, which is also achieved by the tactile‐sensor‐feedback control. When the stable grasping is achieved, the robot lifts the object and transports it to the target location. During this movement, due to the object's weight and the moving disturbances, the object may slip from the robotic hand, possibly leading to task failure. The Hinst robot leverages the real‐time tactile sense to detect the slip, and incrementally increases the grip force to ensure the stable grasping (i.e., stop the slipping). After transporting the object to the target location, the robot proceeds to the third step, which involves executing specific task operation. During this operation, the robot adjusts the grip force based on predefined characteristic criteria to complete the specific operational actions. At last, the robot releases the object and places it at the target location. This task step also requires the robot to adopt the touch‐sense‐feedback to complete the release motion smoothly, especially for the task of releasing an object from a tool.

Take the example of a robot using tongs to pick up a piece of bread and place it onto a plate. A robot hand picks up the tongs stably, and moves to approach the bread. Then, the robot clamps the bread with the tongs, delivers it to the plate location, and finally releases the bread onto the plate. When the robot grasps the tongs, it is essential to keep a firm grip but with as small force as possible to avoid the tongs too tight that would hinder grabbing the bread in the subsequent step. Afterward, the robot adopts the delicate tactile sense to control the process of clamping the bread. In the step of releasing bread, the robot loosens its grip to allow the bread to be smoothly placed down meanwhile keeps the tongs not falling from the robotic hand by the assistance of the tactile sense. The Hinst with touch‐sense‐feedback control ensures the success of the entire process using tongs.

### 2.2 The Contact Cognition and Control Strategy of Robotic Hinst

**Figure 3:**
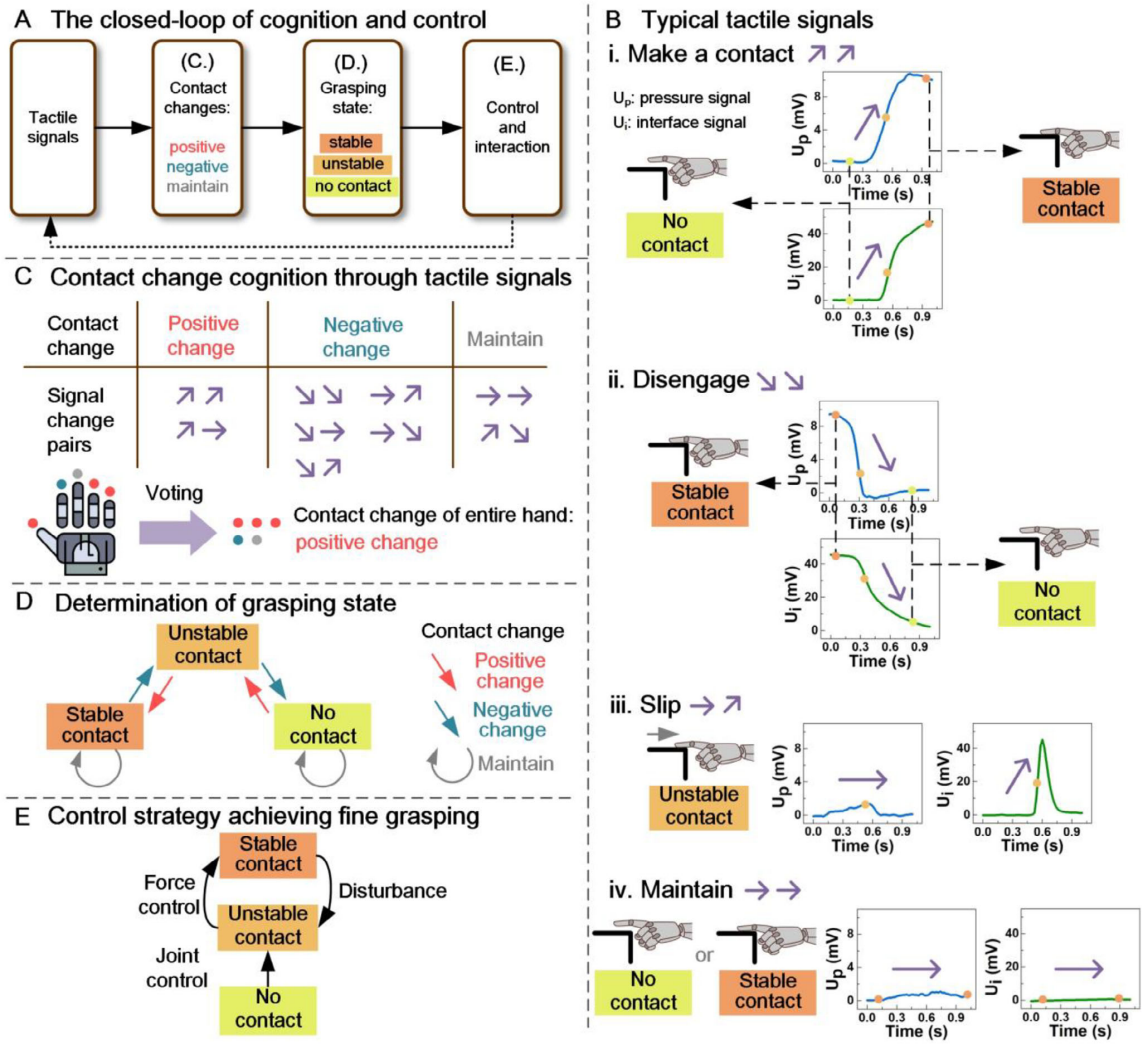
The contact cognition strategy and control strategy of robotic interactive instinct. A) The closed‐loop of contact cognition and control. B) Typical tactile signals: contact(i), disengage(ii), slip(iii), and maintain(iv). C) Contact change cognition through tactile signals and voting. D) Determination of grasping state through contact change. E) Control strategy achieving fine grasping.

As mentioned before, due to complex interactive processes during manipulation, effectively cognizing interactive states and executing interaction control are generally difficult. To address this issue, we propose a general‐purpose knowledge/logical reasoning cognition method. **Figure** **3**A illustrates the contact cognition and closed‐loop control strategy of robotic interactive instinct. The closed‐loop control operates at a frequency of 50 Hz. First, we propose a contact change criterion according to the tactile signals of robotic fingers (Figure 3B, details in Section 2.3), which determines whether the current contact change is positive change, negative change, or remains unchanged (Figure 3C). The positive change refers to a trend toward stable contact. Conversely, the negative change refers to the large probability of slipping or disengaging. Maintaining state refers to the finger maintaining either stable contact or no contact with the object. Then the robot employs a vote strategy to fuse the tactile signals of all fingertips to figure out the contact change of the grasp (Details in Experimental Section). Afterward, the contact change is further converted to robotic grasping state, by combining the current contact change with the previous interactive state to determine the current grasping state (Figure 3D). The grasping state triggers a robotic control adjustment (Figure 3E), i.e., executing adaptive manipulation. This contact cognition and closed‐loop control do not require the involvement of high‐level decision‐making, enabling swift instinctive interaction.

Four typical tactile signals are categorized for the contact process: making a contact, disengaging, slipping, and maintaining (Figure 3B). The tactile signals come from two channels: interface signals and pressure signals. Pressure signals reflect the magnitude of grasping pressure. Interface signals respond to the state change at the contact interface, e.g., touch and slip. When the robotic fingers equipped with tactile sensors come into contact with an object (Figure 3B, i), both interface and pressure signals become increasing and tend toward stability. As a finger disengages from the object, the signal changes are the reverse of the contact process, that is decreasing to no contact state (Figure 3B, ii). When a slip event occurs, the interface signal increases significantly; meanwhile the pressure signal fluctuates moderately (Figure 3B, iii). If the finger stably contacts the object or no contact, both interface and pressure signals remain unchanged (Figure 3B, iv). This contact cognition is universally applicable to all robotic object manipulations.

In the real‐world scenarios, various complex conditions may be encountered, such as uneven object surfaces, non‐ideal grasping angle or position. To simplify the contact cognition across complex real‐world scenarios, we propose the knowledge reasoning and training‐free feature extraction method from the tactile signals of robotic hand. Since the signal changes provide the indications of the contact variations, we extract the characteristic changes of tactile signals as the indicators of grasping state. The details of extracting and judging methods are in the Experimental Section. We denote the changes of pressure and interface signals as a signal change pair. Based on the physical principles of interface and pressure sensing, we categorize all signal changes into three contact states: positive change, negative change, and maintaining state. The robot utilizes a voting strategy to fuse the signals of all fingers to determine the contact change of current grasp (Figure 3C). Then the state transition strategy is employed to determine the grasping state (Figure 3D). Finally, the robot executes a force or joint adaptive‐control to achieve a stable contact throughout the manipulation (Figure 3E and Figure 2B). The contact cognition and control of Hinst are operated with a lightweight logical reasoning algorithm (computation latency <0.1 µs), allowing the robotic hand swift adjustments and timely reactions. In addition, this contact cognition and control strategy as an embedded innate capability are applicable to various interaction scenarios and allows robot universal manipulations. Even if the task is failed, the robot autonomously retries the manipulation by adjusting the grasp force until the task is accomplished. Details are in the Experimental Section.

**Figure 4:**
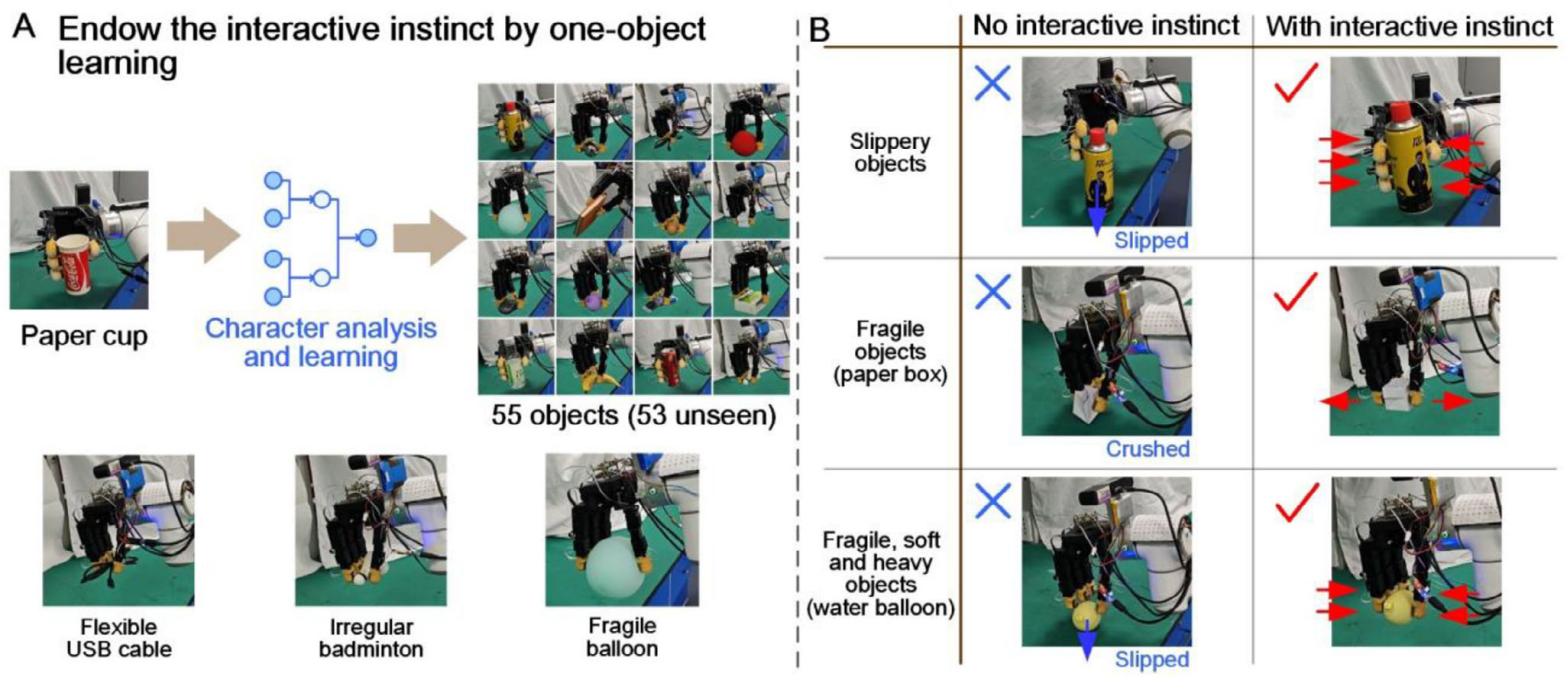
Validation of the proposed robotic interactive instinct. A) Endow robot the interactive instinct to stably grasp various objects by one‐object learning. B) The comparison between the grasping tasks with and without interactive instinct.

To validate the generalizability and universality of the Hinst in robotic manipulations, we conduct an experiment where the robot is empowered with the Hinst via data‐efficient one‐object learning of picking up paper cups (empty and filled with water) and then the Hinst robot can pick up 55 different objects (**Figure** **4**A). These 55 objects cover diverse attributes, including different surface textures (smooth/rough), different hardness/softness, different material, different shape, size, weight, and fragility. Among them, the robotic Hinst is successfully generalized to pick up 53 other objects that the robot has never seen before, including flexible cable, irregular badminton, and fragile balloon (details in Experimental Section and Movie S2, Supporting Information). The Hinst‐equipped robot takes 5–7 s to pick up each object. The success rate of grasping reaches 97.2%, demonstrating the great generalization and robustness of the robotic Hinst.

Figure 4B demonstrates the comparison between the robots with and without Hinst in picking up some objects, including slippery, fragile, soft, and heavy objects. In non‐Hinst method, the robot employs a pre‐defined force strategy to pick up the objects. Without the Hinst, the robot is unable to autonomously adjust the grip force, leading to slip or damage of the objects. For example, the water balloon slips from the hand, and the paper box is crushed during the grasping process. The robot without the Hinst exhibits a lower success rate of 68.0%, particularly fails in most challenging tasks for slippery and heavy objects. In contrast, the robot with the Hinst can robustly pick up all objects (Movie S3, Supporting Information).

The Hinst utilizes a lightweight knowledge/logical reasoning method instead of a large‐data‐driven algorithm, offering advantages in generality and high efficiency. For example, slippage of an object relative to the robotic hand during task execution indicates an unstable grasping state, prompting the robot to swiftly adjust its grasp. This strategy is universally applicable to all manipulations and can be deployed in robots akin to human instincts. Additionally, the knowledge/logical reasoning algorithm is highly efficient, as demonstrated in Figure 4A. Empowering the robot with Hinst merely requires data‐efficient one‐object learning, then the robot can manipulate 55 objects (53 unseen) with a high success rate of 97.2%, including those that are slippery, fragile, or soft and heavy.

Table S1 (Supporting Information) presents a comparison between the proposed Hinst method and other existing methods in object manipulation tasks, bolstering the superiority of Hinst in terms of generalizability, efficiency, and robustness.

### 2.3 Hinst Assisted Robotic Learning Intelligence and Practicing Intelligence

**Figure 5:**
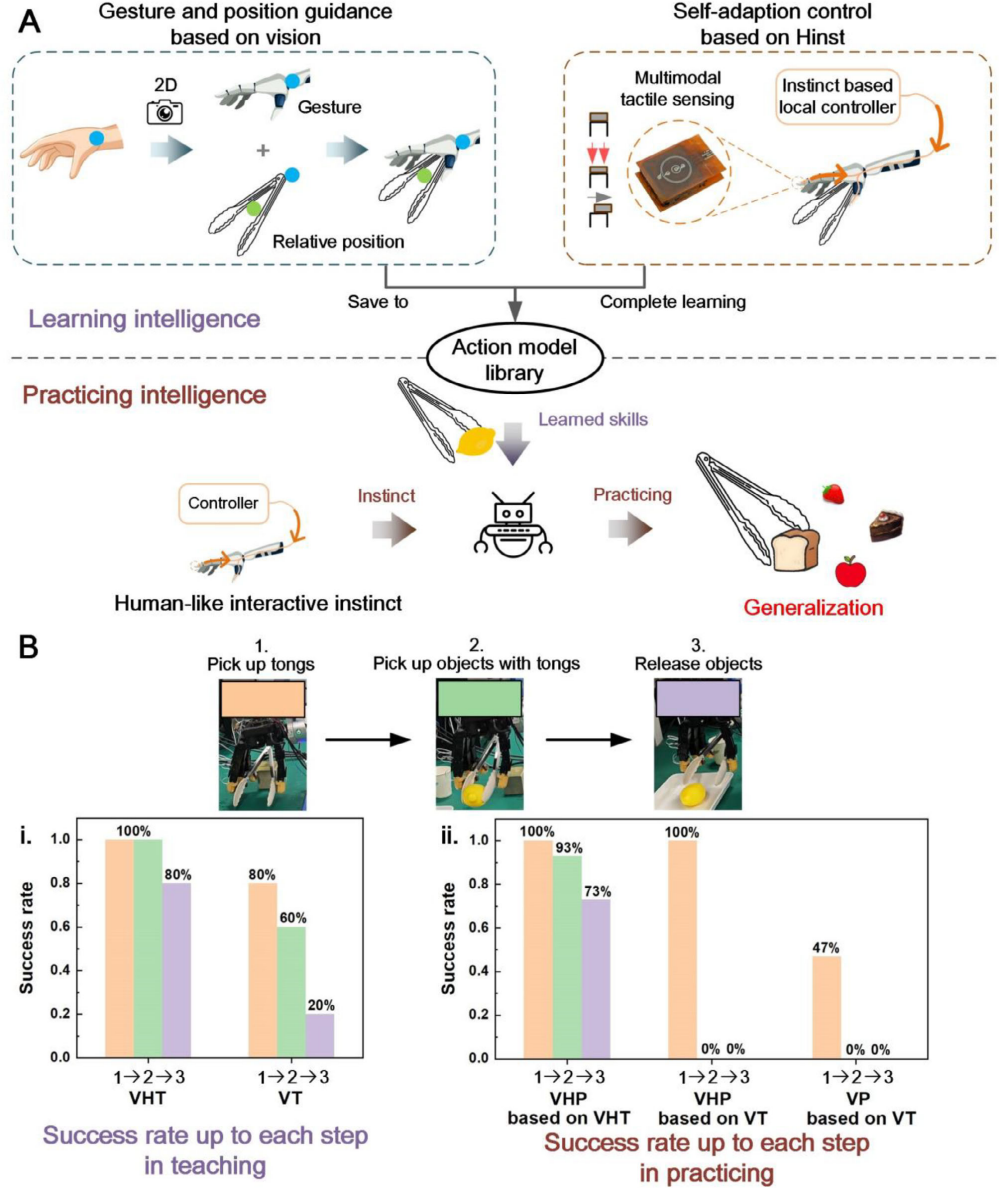
Robotic learning intelligence, practicing intelligence, and the comparison of success rates between the tasks with and without Hinst. A) Learning intelligence: with a 2D camera to watch human guidance, combined with the robotic instinct, the Hinst robot learns the manipulation skills by one‐shot learning. Practicing intelligence: with the skills as guidance, combined with the robotic instinct, the Hinst robot autonomously adjusts its gestures and grip force in its practice, ensuring a robustness, adaptability, and strong generalization. B) (i.) The comparison of teaching success rates with and without Hinst. The task is teaching the robot to use the tongs to pick up an object and place it onto a tray. (ii.) The comparison of robotic practice success rates with and without Hinst.

Robots acquiring manipulation skills from human demonstration is essential for expanding robotic capability, but should be easily accessible and cost‐efficient in practical application. Current robot skill learning mainly relies on vision‐based imitation learning. However, this method faces significant challenges in transferring skills from humans to robots due to the difference in the structure and function of the ontology between humans and robots. Here, we propose a one‐shot learning method incorporating vision with robotic interactive instinct. A low‐cost 2D camera is utilized to capture human's action in one‐shot demonstration. And then, the robot follows along the human's movement (e.g., hand gesture); meanwhile the robotic interactive instinct assists in accomplishing the manipulation task (**Figure** **5**A). After the one‐shot learning, the robot learns the gesture and records the gesture data in the action model library as action primitive for subsequent uses.

During the one‐shot learning, humans can verbally communicate the task requirement to the robot, and can also employ an RGB‐D camera incorporating with VLMs to locate the target objects. The human's movements captured by the camera provide a reference for the robot manipulation. The robot does not need to exactly copy human's movement, instead, the embedded robotic interactive instinct acts as a leading role to accomplish the manipulation (Figure 5A). In other words, the robot references human's gestures to grasp the object; meanwhile, the contact cognition and adaptive control of robotic interactive instinct are taken initiative to accomplish the learning process. The learned manipulation skill is recorded in the robotic action model library for subsequent uses. More details are in the Experimental Section.

In the real‐world robotic tasks, the success of manipulation is not dominated by the exactness of robots imitating human's movement, but rather by robotic delicate sense and rapid control of interactive instinct in robot practice. Our proposed Hinst renders robot a practice intelligence, empowers its self‐adaptability via trial‐and‐error in the interaction practice. When the taught skills need to be executed, the robot references the taught gestures. Based on the contact cognition, the Hinst enables the robot to autonomously adjust its gestures and grip force during the interaction, thereby accomplishing the manipulation task. Since the Hinst is based on universal interaction practice from a general‐purpose knowledge reasoning rather than data‐driven modeling, it can be generalized to unseen objects across multiple scenarios. For instance, after robot learns to use tongs to pick up a lemon, it can generalize to pick up a bread, strawberry, cake, etc.

To validate the generalizability and the practicality of robotic learning and practice intelligence with the Hinst, we conduct a comparison experiment (Figure 5B and Movie S4, Supporting Information). A human teaches a robot to use tongs to pick up and release an object. A human demonstrates the task, and a robot learns the manipulation task by vision only (denoted as VT), or combination of vision and tactile‐sense‐based Hinst (denoted as VHT). In VT, the robot adopts a 2D camera to capture human's gesture and movement, and then follows along the action. In VHT, the robot captures human's gesture and movement by the camera, and follows along the action meanwhile utilizing the Hinst to execute the manipulation task. After the skill learning, the robot practices the task, with vision only (denoted as VP), or combination of vision and Hinst (denoted as VHP). We evaluate the significance of the Hinst by the success rates in both teaching and practicing. The results are shown in Figure 5B.

The first metric is the success rate of teaching. We conduct five teaching experiments where a robot is taught to use tongs to pick up and release a lemon using VT and VHT respectively, and the success rates up to each step are shown in Figure 5Bi. The manipulation task involves three steps (as shown in Figure 2B): pick up the tongs, pick up the object with the tongs, and release the object from the tongs. It is seen that the VT reaches a success rate of 20% for entire manipulation task, while the VHT achieves a high success rate of 80%. The success rates of VHT up to the three steps (100% for picking up the tongs, 100% up to picking up the object with the tongs, and 80% for completing the entire teaching) are 25%, 67%, and 300% higher, respectively, compared to those of VT (80%, 60%, and 20% success rates for VT).

After the teaching with VT and VHT respectively, we further conduct the practice experiments of the robot with VP and VHP respectively. To demonstrate the generalization capability, we select the different objects with different softness and different sizes: strawberry, lemon, and sponge ball, in the practice experiments. The robot practices the picking‐up tasks on each object for five times. Figure 5Bii shows the results of success rate corresponding to three steps. The group of VHP based on VHT reaches the highest success rates across three steps (pick up tongs 100%, pick up object with tongs 93%, release object 73%), indicating the great significance of the Hinst in both teaching and practicing processes. The group of VHP based on VT accomplishes the action of picking up the tongs at success rate of 100%, but fails in picking up the object with the tongs (0% rate), that is because the robot gains null interaction experience in using the tongs to pick up the object. Without the interactive instinct, the robot fails mostly in both learning and practice processes. It is evident that the robotic interactive instinct enhances not only the robustness of robotic learning but also the feasibility of the real‐world practices, especially for the dexterous tasks requiring delicate tactile sense. Instinctual adaptive ability based on touch‐sense‐feedback enables the robot to achieve a high success rate in delicate manipulation, which is necessary for general‐purpose robots. Table S2 (Supporting Information) presents the detailed success rates for picking up each object, indicating that robotic manipulation based on the Hinst can be generalized to various objects with different softness.

### 2.4 Robot Serves Breakfast for Humans

**Figure 6:**
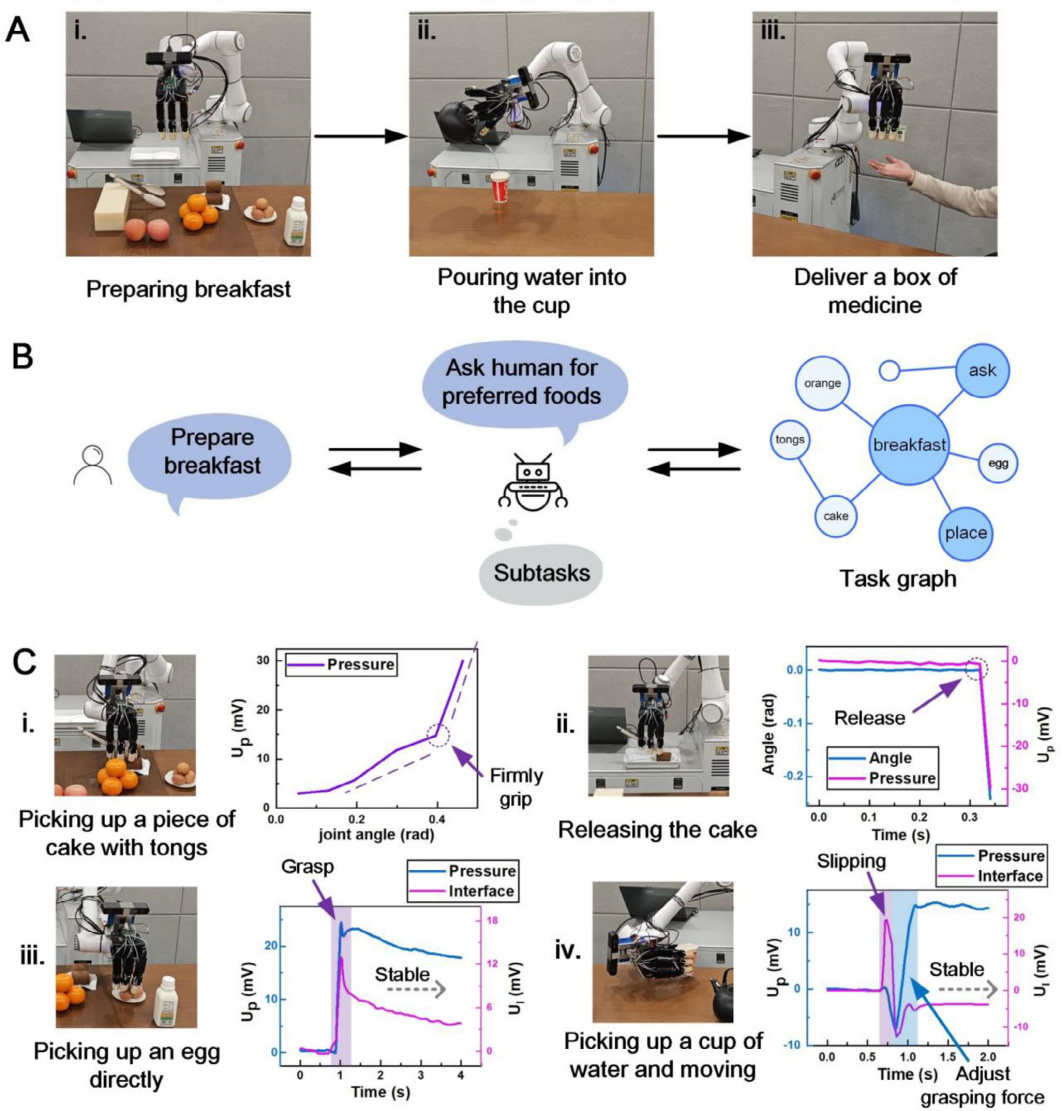
Robotic applications in real‐life scenarios. A) The Hinst robot conducts breakfast service, pouring water into the cup, and delivering a box of medicine. B) Task graph helps robot autonomous task planning according to human preference. C) Tactile sensing graphs of (i) picking up a piece of cake with tongs, (ii) releasing the cake to a tray, (iii) picking up an egg, and (iv) picking up a cup of water and delivering.

To showcase the application potential of the Hinst robot, we apply the robot to a real‐life scenario, where a robot equipped with the Hinst, visual sense, and auditory sense accomplishes a breakfast service for humans (**Figure** **6**A). The tasks contain preparing and transporting foods, pouring water into a cup, delivering a box of medicine to humans (Movie S5, Supporting Information), as well as handing disabled people walk (Movie S6, Supporting Information). In the demonstration, the robot receives human's command, autonomously completes the task planning, then prepares foods and medicine, and finally serves them to the human.

Upon receiving a command of breakfast service, the robot first asks human for his/her preference on foods and makes a task planning based on the task graph locally deployed on the robot to decompose the task into a sequence of executable subtasks: locating, picking up, and transporting the foods to the human (Figure 6B; Figure S2, Supporting Information). In the subtask of picking up the foods, the robot smartly picks up a piece of cake using tongs, and picks up the foods with skins (e.g., egg and orange) using robotic hands directly, achieving meticulous manipulation.

When utilizing the tongs to pick up the cake, the robot combines the tactile sense and the finger joint angle as indicators to exactly determine the moment when the tongs firmly grip the cake and the moment to release it, which has been taught in prior and recorded in the action model library as action primitive. When the tongs make contact with the cake or disengage, there is a significant change in the pressure signal or the ratio of pressure change to angle change, which is used to achieve contact cognition (Figure 6C, i,ii). It is worth mentioning that the robot using tongs to pick up the cake and place it onto a plate is very challenging. When the robot grasps the tongs, it is essential to keep a firm grip but with as small force as possible to avoid the tongs too tight that would hinder grabbing the cake in the subsequent step. Afterward, the robot adopts the delicate tactile sense to control the process of clamping the cake. It needs the robot to precisely perceive the contact state between the tool and the object through tactile sense, and fine control the grasping force. In the step of releasing cake, the robot loosens its grip to allow the cake to be smoothly placed down meanwhile keeps the tongs not falling from the robotic hand by the assistance of the tactile sense. When picking up an egg, the robot perceives its grasping state and controls the grip force to achieve stable grasping (Figure 6C, iii). When picking up a paper cup filled with water, due to the water weight, a slip occurs that triggers the robot to autonomously adjust its grip force to achieve a stable grasping (Figure 6C, iv). Throughout the entire task, the Hinst plays a significant role in facilitating stable and robust manipulations for the robot, demonstrating its universal use and robust practicality. The robot Hinst architecture incorporating learning and practicing intelligence greatly expands the scope of robotic competence and enhances the potential for general‐purpose robots to be applied in the real world. Robots can readily learn skills from humans in a humanized way (through one‐shot demonstration) and practice the skills autonomously, where robotic interactive instinct greatly improves the efficiency and the success.

## 3 Discussion

In this work, inspired by the innate and acquired abilities of humans, we propose a human‐like interactive instinct (Hinst) and the Hinst robotic architecture for general‐purpose robots. The robotic interactive instinct, based on multimodal touch‐sense‐feedback with knowledge‐driven cognition and adaptive control, enabling stable and reliable accomplishment of manipulation, is universally applicable to various tasks in the real world. The foundation of the Hinst architecture lies in the tactile‐sense‐based interactive instinct, ensuring robust manipulation, while the front‐end task planning and action planning modules correspond to the robotic acquired abilities. These innate and acquired capabilities enable the robot to flexibly handle versatile tasks. Our experiments show that the Hinst can be readily empowered to robot by using data‐efficient one‐object learning, and generalized to manipulate 55 objects with a high success rate of 97.2%, including those that are slippery, fragile, or soft and heavy. To further enhance robotic skill learning efficiency, we propose a Hinst‐based learning method that combines robotic interactive instinct with one‐shot human demonstration. Taking a pick‐up task with tongs as an example, we demonstrate how robotic interactive instinct improves its learning efficiency and task success rate. The generalizability of the Hinst allows the robot to extend its learned skills from one object to other objects. Moreover, we showcase a breakfast service task that the Hinst robot successfully accomplishes meticulous housework in the real world.

It is worth noting that the aforementioned experiments of Hinst‐based grasping, skill learning, and task demonstrations (Movies S2, S4–S6, Supporting Information) are demonstrated in different environments (i.e., different rooms) to validate real‐world scalability of the proposed methodology.

Beyond the multimodal tactile sensors used in our system, we believe that other tactile sensors capable of perceiving contact, pressure, and slip can be similarly utilized into the Hinst to endow robots with interactive instinct and task execution capabilities.

Our proposed Hinst demonstrates that the interactive instinct is essential for improving operational robustness and learning efficiency of robots. Future work may focus on diversifying skill acquisition methods. We will explore the use of the Hinst to capture more complicated operation of tools, enabling robots to learn more advanced skills.

The robots with human‐like interactive instincts are expected to pave the way for the applications of general‐purpose robots. Beyond the home applications demonstrated in this work, in the future, we also aim to extend the Hinst robot to accomplish industrial skilled tasks, such as fine assembly and sorting.

## 4 Experimental Section

### Task Planning and Task Graph

To complete task planning and execution for general‐purpose robots, a hierarchical framework was proposed, spanning from the bottom interactive instinct to the top‐level decision‐making, as illustrated in Figure S1 (Supporting Information). Initially, natural language processing (NLP) was utilized to extract verbs and nouns from the abstract human instructions received by the robot. The extracted verbs were matched within the task graph and planned as a set of action primitives from the action model library, while the nouns were matched with objects in the graph. Subsequently, the robot executes the planned actions, during which the human‐like interactive instinct ensures the robustness of the manipulation. If the pre‐deployed task graph does not contain the required task, the robot may seek the assistance from cloud LLMs and VLMs to accomplish task planning.

Additionally, the robot can learn skills from human demonstration and enriches its action model library. LLMs and VLMs may assist the robot in accomplishing simple tasks in a zero‐shot manner, such as sorting objects on a table (Movie S7, Supporting Information). Here, the APIs used for LLMs and VLMs were Qwen‐plus and Qwen‐VL, respectively.

The task graph was constructed using a knowledge graph approach, which was used to obtain the planning rules for tasks. As illustrated in Figure S2 (Supporting Information), the knowledge graph contains two categories of entities: task and object. The planning relationships were used to connect a task with its subtasks, indicating that a task requires the invocation of corresponding subtasks to be realized. Planning relationships from objects to tasks indicates that the task should be executed before manipulating the object. Tool relationships from objects to objects indicate that a specific tool was needed to manipulate the object. For example, in the case of the breakfast service task, the essence of the breakfast task was picking and transporting task. The food selection was inquired from human. Therefore, upon receiving the breakfast task command, the robot asks human for his/her preferred foods, such as cake, egg, and orange. Subsequently, the robot plans the breakfast service task as follows: pick up a piece of cake, an egg, an orange, and then transport them to the human. There was a tool relationship between the object “cake” and the “tongs,” therefore the robot uses tongs to pick up the cake. Neo4j was used to construct the task graph. The task graph can be augmented using LLMs or human's guidance. Python programing was used to accomplish the all of the functions.

### The Structure of the Tactile Sensor

The tactile sensor in this work was based on thermosensation and developed by ourselves, composed of two sensing layers with the same sensing structure.^[^ ^27^, ^30^ ^]^ Each sensitive unit consists of two concentric Pt thermistors deposited on a flexible polyimide substrate (Figure S3, Supporting Information illustrates the structure of tactile sensor). The inner circle had a lower resistance (≈50 Ω) and serves as the heat source, generating a thermal field. The outer circle had a higher resistance (≈500 Ω), and it essentially does not generate heat, maintaining a temperature consistent with the environment (or the contact object). Utilizing a constant temperature difference (CTD) conditioning circuit, the temperature difference between the outer and inner resistances was kept constant, which self‐sustains the temperature‐compensation of the tactile sensor.^[^ ^31^ ^]^ The interface sensor (the top sensing layer) detects the contact states with the object, for example when the object comes into contact with the sensor, or slips, or detaches. The contact change influences the heat transfer of the interface sensor, and thus be detected. For the pressure sensor (the bottom sensing layer), there was a porous material with piezo‐thermic transduction, enabling the measurement of pressure. When pressure was applied to the sensor, the porous material was compressed, further influencing the heat transfer of the pressure sensor, resulting in the detection of pressure. These sensing principles were based on conductive heat transfer, which was universally applicable to all objects.

### Technical Details of Tactile Sensor

The performance of the tactile sensor was tested and shown in Figure S4 (Supporting Information). Figure S4A,B shows the tactile sensor exhibits a good stability against the temperature changes (from 25 to 50 °C) and the humidity changes (from 40% to 70% RH). Figure S4C (Supporting Information) shows the sensor's long‐term stability test, indicating that both the interface signal and pressure signal remain stable under 10 000‐cycles repeatability test. The results demonstrate good stability and durability of the tactile sensor for uncertain environment and long‐term uses.

Figure S4D (Supporting Information) shows the calibration of the pressure sensor. And Figure S4E (Supporting Information) shows the pressure sensor exhibits a lower detection limit of 0.01 N and a response time of 80 ms.

### The Extracting and Voting Method of Contact Change based on Tactile Signals

For both pressure and interface signals, there were three possible changes: increase, decrease, and no significant change. For the tactile sensors that perceive both pressure and interface, they result in nine possible signal change pairs. Based on the sensing principles of the sensors, these nine signal change pairs were categorized into three types of contact changes.

As demonstrated in Figure 3B, when an object comes into contact with the tactile sensor, both the interface and pressure signals increase. If pressure continues to be applied after stable contact was achieved, the pressure sensor signal will continue to increase, while the interface signal will not. These two scenarios were classified as a positive change, indicating that the contact between object and the robotic hand was moving toward a stable state or becoming more stable. Disengaging and slipping were classified as negative changes. During disengaging, both the interface and pressure signals decrease. During slipping, the pressure signal remains unchanged while the interface signal increases. These two signal trends mean that the contact between the object and the robotic hand was shifting toward an unstable state. If there was no timely adjustment, the object may fully slip and result in a failed grasp. Therefore, these two signal changes were considered negative changes. In addition, a situation where the pressure signal decreases while the interface signal remains unchanged was a negative change. It may occur when there was a slight reduction in pressure between the sensor and the object, without any indication of separation. If the pressure continues to decrease, disengaging will occur. Another negative change was when the pressure signal remains unchanged while the interface signal decreases. This signal change may happen due to an uneven surface of the contact object: slipping occurs, while the sensor moves from a region with relatively fine contact to a region with poor contact. Another negative change was the pressure signal decreasing while the interface signal was increasing, which may be caused by the simultaneous occurrence of slipping and disengaging. For the maintain state, the typical situation was when there were no significant changes in both pressure and interface signals. In addition to this, an increase in the pressure signal while the interface signal decreasing was a situation that theoretically should not occur, as there was no reasonable way for the interface signal decreasing while the contact pressure increases. Therefore, it was temporarily classified it as maintain. Figure 3C summarizes all the contact change situations. The acquisition of these parameters for determining increases, decreases, and no significant changes in pressure and interface signals is described in the “Grasping Experiment” section.

In real‐world robotic manipulation, it was usually not possible to ensure good contact between the robotic fingers and the object to be grasped, a voting strategy was proposed to obtain the contact change of the entire hand. In subsequent steps, the contact change of the entire hand was further used to determine the grasping state. The robot had a dexterous hand with four fingers, on the fingertip of which four tactile sensors were equipped respectively. The contact changes obtained from four fingers were voted on. If the number of positive changes was greater than the number of negative changes, the contact change of the entire hand was determined to be positive change; otherwise, it was determined to be negative change. If the numbers were equal, it was determined to be maintain.

### Control and Retry Strategy of Practicing Intelligence

The robotic hand employs a proportional‐derivative (PD) control on its joint angles. When targeting the same joint angles for grasping an object, the output torque of the motors was positively correlated with the proportional coefficient. Therefore, the proportional coefficient was approximated as the parameter to control the robotic grasping force. Two grasping parameters were designed to achieve force control in task manipulation: the initial grasping force and the step size of force increase. These two force parameters correspond to a relatively small initial proportional coefficient and a proportional coefficient adjustment step size, respectively. Given the various contacts between the sensors and objects in the real world, the use of pressure measured by sensors to assess the robotic grasping force was excluded.

Figures 2B and 3E illustrate the control strategy. The control strategy from no contact to stable contact: the robot begins with the initial grasping force, then increasing the grasping force while continuously monitoring contact changes. If the tactile sense infers a sustained positive change lasting 0.1s, the robot determines that a stable grasp had been achieved; otherwise, it remains in a transitional unstable state and continues to increase the grasping force. The basis for the criteria is provided in Experimental Section. The feedback control strategy for maintaining stable contact: if a negative change was detected in stable state, which was considered the onset of unstable state, the robot immediately increases the grasping force to prevent the worse of the unstable state. The judgment strategy for no contact: if the pressure returns to the baseline, the grasping state was considered to had reverted to no contact. An unexpected no contact state during a task indicates that the object had slipped, and the task fails.

Task failure indicates that the adopted control strategy was no longer applicable to the current task. Therefore, a retry strategy for grasping tasks was proposed. After the task fails, the robot autonomously increases the proportional coefficient of the initial grasping force and the step size of the proportional coefficient adjustment by 20%, and attempts the task again until success.

### Grasping Experiment

The robot conducts four pickups of a paper cup with same grasping configuration. In each pickup, the robot first grasps the cup on the table, then lifts it, and finally releases the cup to attain the tactile signals of disengagement. Two of the four pickups involve empty cups, while the other two had 1/3 of the cup filled with water to induce slipping during lifting.

For contact changes cognition, there were three important parameters: pressure threshold Uthresh1 (the threshold for determining whether the pressure signal had increased or decreased), interface threshold Uthresh2 and the reference for judging the current contact change. From the experiments, the changes in tactile signals (pressure and interface) during contact, disengagement, slipping, and the noise of tactile signals in the maintain state were extracted (Figure S5A, Supporting Information). The objective of solving for pressure threshold Uthresh1 was to find a threshold value that can distinctly differentiate between signal changes due to contact/ disengagement/slipping and interference noise as pronounced as possible (similar for interface threshold Uthresh2). For the problem of determining the threshold in a 1D space, a support vector machine (SVM) with a linear kernel was employed to solve for Uthresh1 and Uthresh2 separately (Figure S5B, Supporting Information). We do not need to collect large data because the sensors provide direct and sensitive measurements of physical quantities including slipping and contact. In fact, as can be seen from Figure S5B (Supporting Information), the noise was significantly smaller than the signal changes caused by contact and slipping. Moreover, both the pressure and interface thresholds were greater than 3σ noise level of the no contact state. To determine the contact change of the current signal, it was necessary to take a segment of the previous signal as a reference. During the contact process, considering that the spikes in the signal's rising phase may interfere with the judgment and the rise time was ≈0.2 s, the reference value for each moment is the mean of the signals’ value in the interval 0.4–0.2 s prior.

The criterion for stable contact determination: based on the obtained Uthresh1 and Uthresh2, and combining the four experiments, the signal can rise above the threshold within 0.1s from the onset of contact. Therefore, it was considered that when there was consecutive 0.1 s positive changes represented the transition of the grasping state from no contact/unstable contact to stable contact.

### The 55 Objects in Pick‐Up Experiment

Prior‐unseen 53 objects were used in the pick‐up experiment: Banana, mango, orange, jujube, egg, strawberry (model), carrot (model), apple (model), grape (model), milk box, toothpaste, gloves, medicine bottle (large and small), metal bottle, sponge ball (large and small), metal ball, paper ball, notebook, medicine box, computer mouse, headphone case, power adapter, eyeglass case, pencil case, USB data cable, flat box, paper box, plastic cup, hand soap bottle, hand cream, balloon, water balloon, bag, stapler, tape, plastic bottle, pack of facial tissues (large and small), can, badminton shuttlecock, ping‐pong ball, tennis ball, baseball, bowl, toy dinosaur, toy tire, toy car, doll, building blocks (star‐shaped, arch‐shaped, triangular).

Two objects were used to endow the robot with the Hinst via a one‐object learning: paper cup (empty, filled with water).

Slippery objects with smooth surface: metal bottle, metal ball, can, egg, toothpaste.

Objects with rough surface: toy dinosaur, tennis ball, paper ball, doll, eyeglass case.

Hard objects: metal bottle, metal ball, strawberry (model), apple (model), grape (model), medicine bottle, building blocks, toy car.

Soft objects: paper box, sponge ball, water balloon, carrot (model), gloves, pencil case, USB data cable, plastic cup, doll.

Objects with irregular shape: badminton shuttlecock, power adapter, USB data cable, computer mouse, hand cream, stapler, bowl, toy dinosaur, building blocks (star‐shaped), banana.

Relatively large‐sized objects: metal bottle, medicine bottle (large), sponge ball (small), hand soap bottle, balloon, pack of facial tissues (large).

Relatively small‐sized objects: jujube, sponge ball (small), headphone case, ping‐pong ball.

Relatively heavy objects: water balloon, metal bottle, paper cup (filled with water), metal ball, hand soap bottle, hand cream, milk box (filled with milk).

Fragile objects: balloon, water balloon, badminton shuttlecock, egg, paper cup, paper box, banana, mango, plastic cup.

### Visual Guidance and Self‐Adaptation Method of Teaching

To capture human hand gestures and poses by vision, the Mediapipe Hands pipeline was employed.^[^ ^32^ ^]^ This pipeline enables estimation of the pixel coordinates and 3D coordinates (in the intrinsic hand coordinate system) of 21 key‐points on the hand from RGB video frames. Subsequently, the EPnP (Efficient Perspective‐n‐Point) algorithm was utilized to solve the PnP (Perspective‐n‐Point) problem, obtaining the transformation matrix from the hand coordinate system to the camera coordinate system. It allows to derive the coordinates of the hand's key‐points in the camera coordinate system. Notably, this method requires only a low‐cost RGB camera, capturing images at a frequency of 30 Hz to estimate hand gestures and poses. This approach provides an efficient and cost‐effective solution for robotic skill learning by human's hand‐by‐hand teaching.

Smoothing filters were applied to enhance motion smoothness in imitation learning. However, due to the limited accuracy and stability of finger joint detection, the joint angle information obtained through visual methods, when directly used to guide finger movements, may lead to tremble in the robotic hand. To solve this problem, a fuzzy logic‐based method was employed to classify finger postures into three distinct states: relaxed, picking, and forceful grasping—based on detected finger joint angles. These states serve as input signals representing human intent. Correspondingly, robotic finger commands were categorized into three actions: relaxation, picking, and forceful grasping. If the human finger transitions from relaxed state to picking posture, the robotic finger initiates a picking action. Subsequently, if the gesture shifts to forceful grasping, the robotic finger increases its grip force; if the gesture remains in the picking state, the grip force was maintained. If the input reverts to relaxation, the robotic hand gradually reduces its grip force until it releases the object and returns to the relaxed state. If the input gesture changes to picking during this process, the grip force was maintained, enabling precise control of grasping force during teaching. A vision‐only teaching approach (denoted as VT) relies solely on the visual guidance for teaching.

For the combined vision and Hinst teaching (VHT) approach, gesture and position guidance methods were enhanced by incorporating Hinst to achieve self‐adaptation in hand operations. Upon receiving gesture intent, the robot can execute control for finger relaxation, picking, and forceful actions with robotic tactile feedback. In the picking state, the robot achieves stable grasping automatically. In the forceful grasping state, the robot uses pressure‐angle feedback (details in the “The characteristic criterion of executing operation” section), increasing the grip force while dynamically assessing whether the appropriate force had been applied. If the human gesture transitions from a forceful grasping gesture to a relaxing gesture, the robot executes a controlled release, such as releasing an object held by the tongs.

### Robotic Skill Learning Method

Prior to teaching, the robot employs an RGBD camera to locate the target objects. In human demonstration, the trajectories, fingers’ joint information, and grip force were recorded for skill learning. Based on the recorded data, regions with significant changes in finger gestures were identified, indicating that these regions correspond to important action operations executed by the robot. The corresponding operational positions of the robot were localized as key points, and the corresponding gestures were marked as key gestures. The key‐points’ positions were aligned with the objects’ positions. The relative positions, key gestures and the skill name were included in robotic action model library for future reference.

### The Characteristic Criterion of Executing Operation

During the execution of operations, if the robotic hand was in a stable grasping state, the contact cognition strategy depicted in Figure 3 is insufficient to assist the robot in implementing dexterous manipulations, since the sensor and object may remain in constant contact. To achieve delicate perception and fast control in robotic manipulation, the pressure on the fingers and the joint angles were regarded as key parameters, which reflect the motion of the hand gestures and the tactile feedback during manipulation.

To obtain the characteristic criterion of execution, the VT method was employed to conduct experiments, where human guides the robot to use tongs to grasp fruit models. During this process, the perception data from the tactile sensors, the joint angles of the robotic fingers, and the outcomes of the task were synchronously acquired, plotting pressure‐angle curves (Figure S6, Supporting Information). Figure S6 (Supporting Information) presents the six nodes of the process (Figure S6A, Supporting Information) and three typical outcomes: successful completion of all the steps (Figure S6B, Supporting Information); completion of picking the object but failure to release the object (Figure S6C, Supporting Information); and completion of picking the object but failure to securely hold the tongs during the release step (Figure S6D, Supporting Information). Since the thumb and index finger play the most significant roles in the touch‐sense‐feedback control, the sum of the pressure signals from the thumb and index finger was used as the vertical axis in Figure S6 (Supporting Information), and the sum of the joint angle changes of the thumb and index finger as the horizontal axis. In Figure S6B (Supporting Information), it was seen that when the robotic hand just makes contact with the tongs, the tongs just make contact with the object, or the tongs just release the object, the curve shows a distinct inflection point. In Figure S6C (Supporting Information), because the inflection point for the release process had not yet appeared, the object was not successfully released. In Figure S6D (Supporting Information), the tongs drop from the hand because the release operation was not stopped in time after the inflection point of release appears. The ratio of pressure change to angle change (Δpressure/Δangle) was utilized as the characteristic criterion for perception, enabling robust manipulation of the tongs. This criterion can be understood through the physical meaning of Δpressure/Δangle. A significant increase of the ratio indicates that the robotic hand had make a contact with the object directly or indirectly, while a significant decrease suggests separation. Based on the Δpressure/Δangle data obtained from experiments, a support vector machine (SVM) with a linear kernel was employed to calculate the threshold for determining inflection point, thereby achieving delicate cognition.

### Configuration of the Hinst Robot

Our Hinst robot was comprised of robotic arm (EC66, ELITE Co. Ltd, Suzhou, China), robotic hand (Allegro hand, WONIK ROBOTICS, Seongnam‐si, Korea), binocular depth camera (ZED 2i, Stereolabs Co. Ltd, San Francisco, America), automated guided vehicle (Oasis‐600C, STANDARD Co. Ltd, Shenzhen, China), and self‐developed electronic skin.^[^ ^33^ ^]^

### The Action Model Library and Basic Robotic Primitives

The robotic action model library encompasses a range of basic action primitives, including visual locating, object picking, approaching and hand movement. For the visual locating module, upon inputting the objects to be located, the robot employs its RGB‐D camera to capture the objects. Utilizing the VLMs’ API provided by Qwen‐VL, the robot locates the pixel coordinates of the object within the RGB image and, in conjunction with depth information, determines the object's position in the robotic base coordinate system. Furthermore, leveraging the point cloud data, the robot roughly calculates the object's length, width, and height, categorizing the object's shape into three types: lump‐shaped, cup‐shaped, and flat object. The object picking module was designed to facilitate the picking of objects. For the object picking module, the robot first invokes the visual locating module to ascertain the object's position and posture. Based on the three distinct shapes, the robot employs corresponding appropriate strategies to complete the picking task. For instance, for flat objects, the robot first moves them to the edge of the table before picking them up, while for cup‐shaped objects, robot grasps them from the side. The approaching module was used to control the automated guided vehicle (AGV) car, which was employed when the robot was at a considerable distance from the target location to move closer to the target.

Through learning, the robot can acquire new skills and expand its action model library to handle a broader range of tasks.

## Conflict of Interest

The authors declare no conflict of interest.

## Author Contributions

R.Z. and Z.J.L. contributed to conceptualization. Z.J.L. and R.Z. developed the methodology. Z.J.L., Q.M., and Y.C.Q. conducted the investigation. R.Z. managed the project. Z.J.L. and Y.C.Q. developed the software. Z.J.L., R.Z., and J.F.Y. performed the visualization. Z.J.L. wrote the original draft. R.Z., Q.M., and Z.J.L. reviewed and edited the manuscript.

## Supporting information



Supporting Information

Supplemental Movie 1

Supplemental Movie 2

Supplemental Movie 3

Supplemental Movie 4

Supplemental Movie 5

Supplemental Movie 6

Supplemental Movie 7

## Data Availability

The data that support the findings of this study are available in the supplementary material of this article.
