# Supplementary material for: Hinst: Human‐Like Interactive Instinct Enables Robots to Robustly Accomplish Universal Tasks
Source: Adv Sci (Weinh). 2025 Jul 28;12(39):e09483. doi: 10.1002/advs.202509483 (PMC12533381; doi:10.1002/advs.202509483)
Supplement: Supplementary file 1 — Supporting Information [file ADVS-12-e09483-s005.docx]

Supplementary Information for

**Hinst: human-like interactive instinct enables robots to robustly accomplish universal tasks**

Zijian Liao^1^, Qian Mao^1^, Yichen Qin^1^, Jinfeng Yuan^1^ and Rong Zhu^1^*

Corresponding author: zr_gloria@mail.tsinghua.edu.cn

**The PDF file includes:**

Figs. S1 to S6

Tables S1, S2

Legends for movies S1 to S7

References (*19, 24-26, 33*)

**Other Supplementary Materials for this manuscript include the following:**

Movies S1 to S7

Fig. S1.


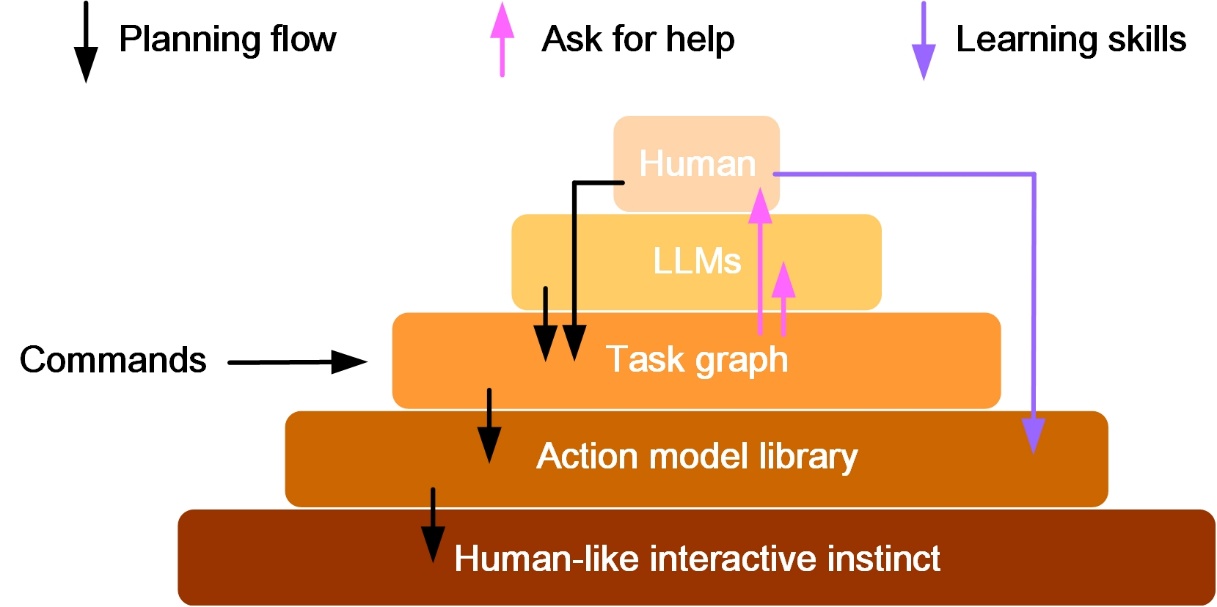


**Fig. S1. The hierarchical framework for general-purpose robotic task accomplishment.** The framework facilitates the transition from the task decision-making to the interaction with objects. Generally, the upper modules guide the lower modules, with the planning flow proceeding from top to bottom. The bottom three layers represent the robot's inherent capabilities, achieving a high degree of automation. When the task graph is unable to complete planning, the robot seeks assistance from the upper modules. The robot can also learn skills from human to enrich its action model library.

Fig. S2.


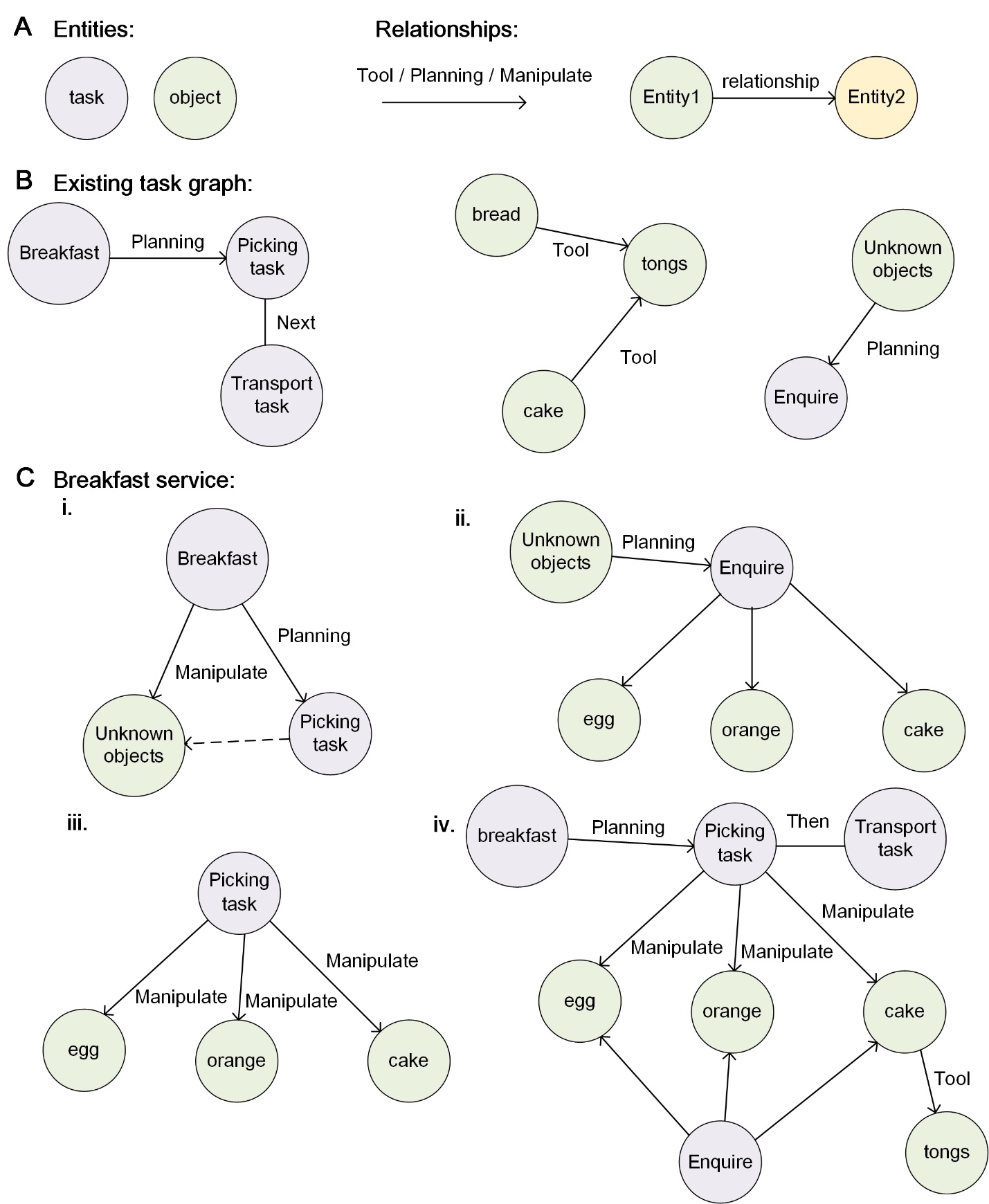


**Fig. S2. The Task graph for task planning.** The task graph is used to obtain the planning rules for tasks. (**A**) Entities and relationships in the knowledge graph. (**B**) Knowledge utilized in the breakfast service. (**C**) Based on the task graph, planning for the breakfast service task.

Fig. S3.


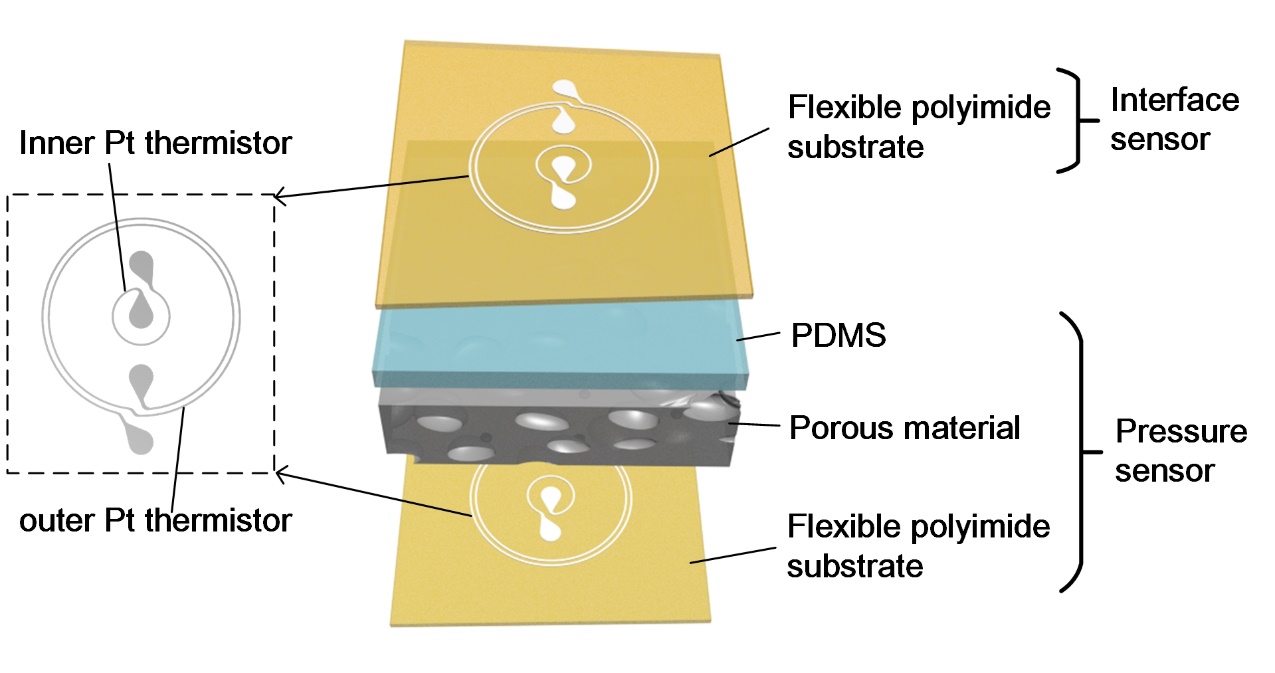


**Fig. S3. The structure of tactile sensor.** The tactile sensor is composed of two sensing layers with the same concentric Pt thermistor structure. The top layer is interface sensor and the bottom layer is pressure sensor.

Fig. S4.

**
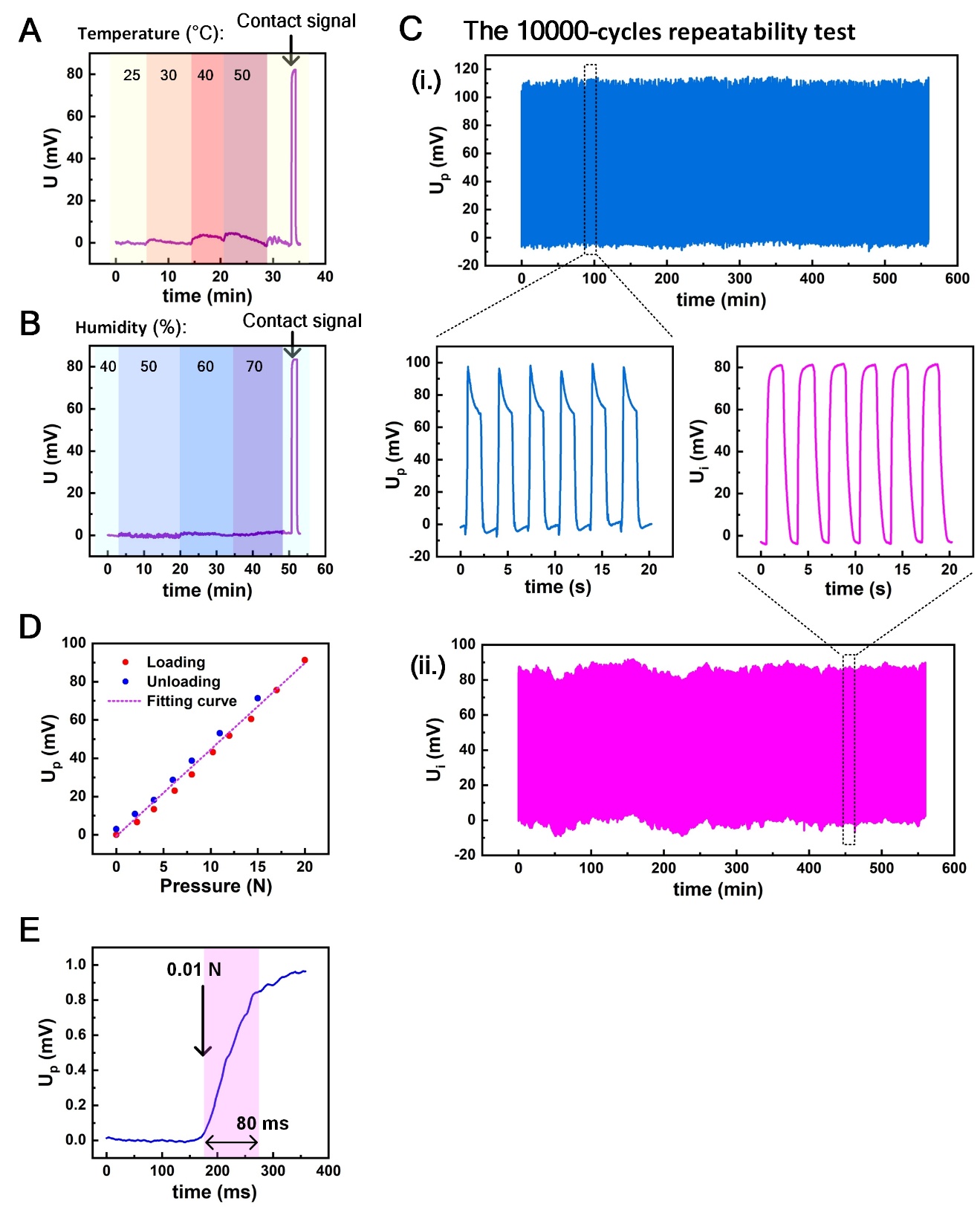
**

**Fig. S4. The technical details of tactile sensor.** (**A**) The performance of tactile sensor under dynamic temperature variations, and the comparison between the sensor's temperature-induced response and the signal generated by a contact. (**B**) The performance of tactile sensor under dynamic humidity variations, and the comparison between the humidity-induced response and the signal generated by a contact. (**C**) The long-term stability of the tactile sensor under 10,000-cycles repeatability test (0-15 N force). (**D**) The calibration of pressure sensor. (**E**) The lower detection limit and response time of pressure sensor.

Fig. S5.


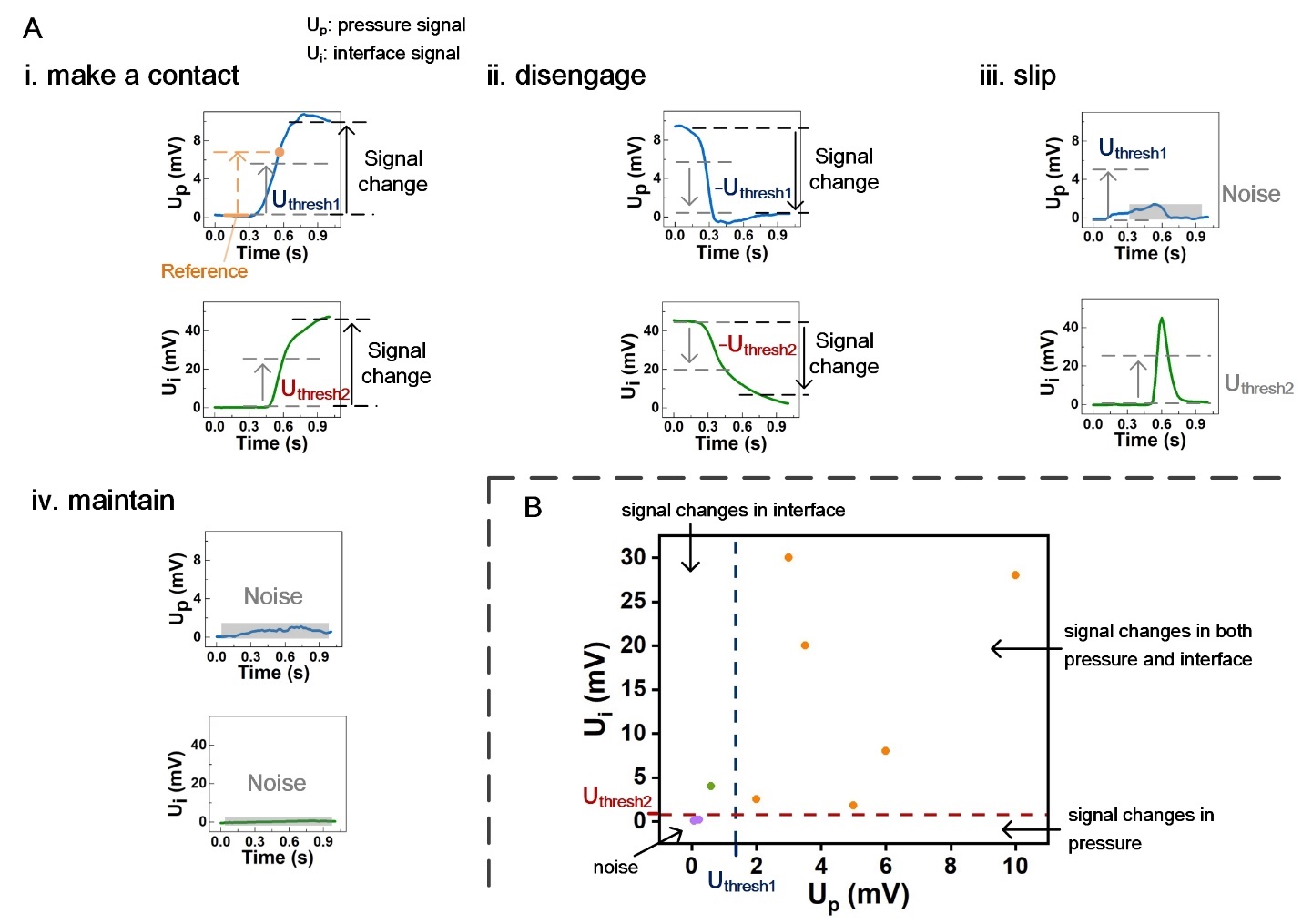


**Fig. S3. Acquiring pressure threshold *U_thresh_*_1_ and interface threshold *U_thresh_*_2_ from tactile signals.** (**A**) Extracting the signal changes and noise from the four typical tactile signals. When making a contact or disengaging (i.) (ii.), both interface and pressure signals exhibit significant changes. When slipping (iii.), interface signal exhibits significant changes and the minor fluctuations in pressure signal can be considered as noise. During maintain state (iv.), the fluctuations in both pressure and interface signals can be considered as noise. (**B**) Utilizing data collected from grasping experiments, *U_thresh_*_1_ and *U_thresh_*_2_ are independently solved using the SVM method to maximally distinguish between the amplitudes of noise and signal changes.

Fig. S6.


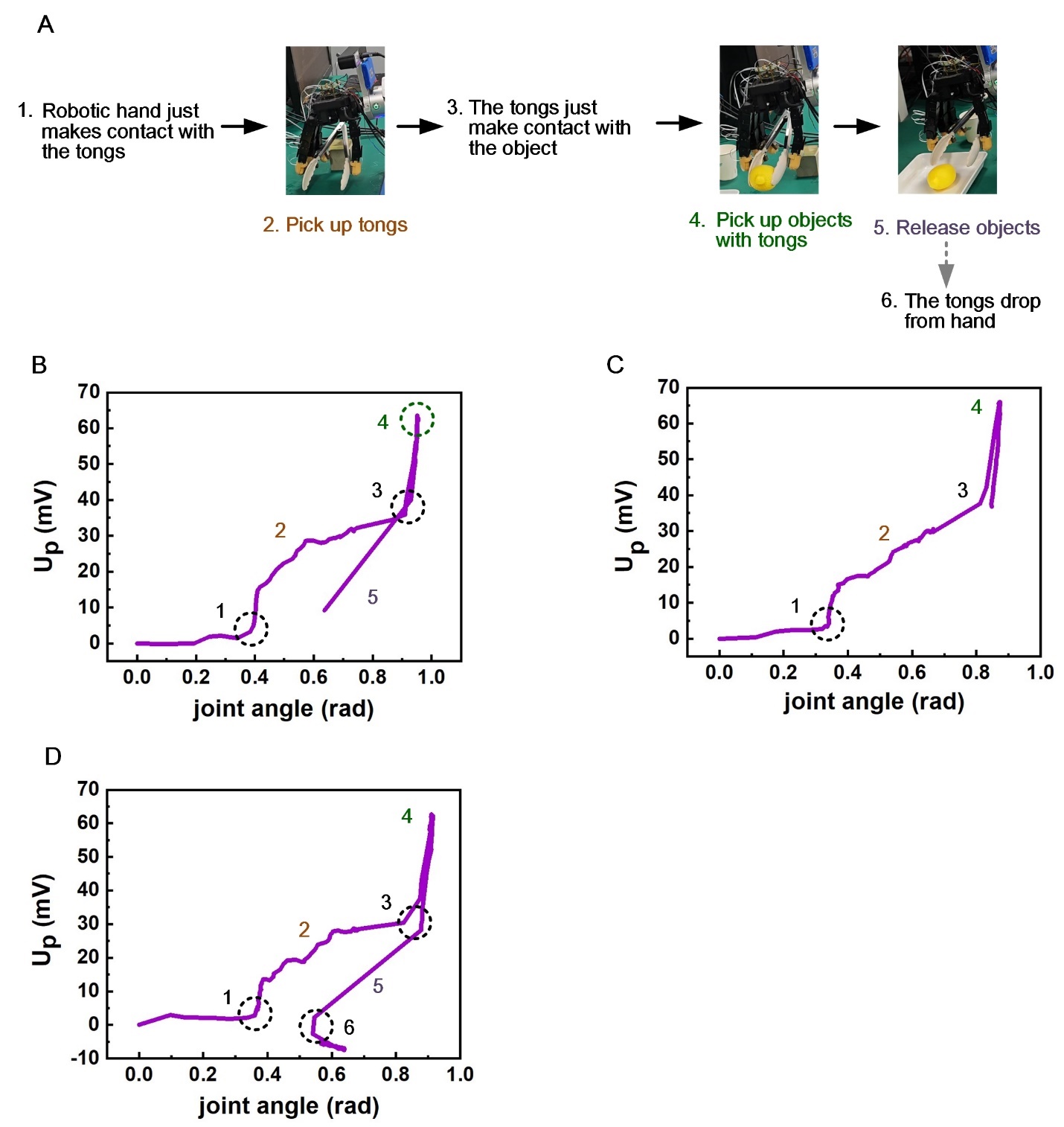


**Fig. S4. The pressure-angle relationship serves as the characteristic criterion for executing operation, with its curves illustrating three typical outcomes.** (**A**) The six key nodes in the process of using tongs to pick up object. (**B**) Curve of completing all the steps. (**C**) Curve of picking the object successfully but failure to release the object. (**D**) Curve of picking the object successfully but failure to securely hold the tongs during the release step.

Table S1.

| Learning algorithm | Transformer (*24*) | Gibbs distribution model (*25*) | SVC and ensemble learning (*26*) | TCN (Temporal Convolutional Network) (*19*) | **Hinst (knowledge reasoning) proposed in this work** |
| --- | --- | --- | --- | --- | --- |
| Tactile sensing type | Vision-based sensor (GelSight) | Vision-based sensors | Piezoresistive sensors | Barometric sensors | **Thermosensation-based sensor (our homemade)** |
| Sensing modality | Slip  (vision-based) | Geometric morphology and pressure (vision-based) | Tangential force and normal force | Pressure, slip (pressure-based) | **Contact, Pressure, slip (independent)** |
| Number of training samples | 5 objects, 782 grasps | 1800 grasps | 6 objects, 300 grasps | 3 objects, 78 slipping modes | **2 objects, 4 grasps** |
| Challenging object attributes | Fragile fruit | Heavy objects, slender objects, irregular objects | Slippery objects, Fragile objects | Slippery objects | **Slippery objects, fragile objects, heavy objects, irregular objects** |
| Number of manipulated objects | 8 | 23 | 6 | 6 | **55 (53 unseen)** |
| Real-world pickup success rate | 88.4% (seen),  60% (unseen) | / | 98% | 72.5% | **97.2%** |

**Table S1. The comparison between the proposed Hinst method and other existing tactile-based/data-driven approaches in object manipulation tasks.**

Table S2.

| Objects | Description | | Method | | |
| --- | --- | --- | --- | --- | --- |
|  |  |  | VHP + VHT | VHP + VT | VP + VT |
| Lemon | Success rates up to each step | 1 | 100% | 100% | 60% |
|  |  | 2 | 100% | 0% | 0% |
|  |  | 3 | 80% | 0% | 0% |
| Strawberry | Success rates up to each step | 1 | 100% | 100% | 40% |
|  |  | 2 | 80% | 0% | 0% |
|  |  | 3 | 80% | 0% | 0% |
| Sponge ball | Success rates up to each step | 1 | 100% | 100% | 40% |
|  |  | 2 | 100% | 0% | 0% |
|  |  | 3 | 60% | 0% | 0% |

**Table S2. The success rates in picking and releasing three objects using tongs, based on three combinations of learning and practicing methods.** We teach robot to execute pick-and-place tasks with tongs using vision-only teaching (VT) and combination of vision and Hinst (VHT) respectively. Then, conducting the practicing experiments with three groups: practice combination of vision and Hinst (VHP) based on VHT (VHP + VHT), VHP based on VT (VHP + VT) and VP based on VT (VP + VT). The results of success rate corresponding to three steps are shown in the table.

Supplementary Movies

Movie S1. Summary of Hinst architecture for real-world task accomplishment.

We introduce the Hinst architecture and demonstrate the skill learning and task accomplishment capabilities of the Hinst robot in the real world.

Movie S2. Picking up 55 objects.

Through a one-object learning involving picking up paper cups (empty and filled with water), the Hinst robot can pick up 55 different objects, including 53 prior-unseen objects.

Movie S3. Comparison between the grasping tasks with and without interactive instinct.

With the Hinst, the robot can pick up various challenging objects, including slippery, fragile, soft, and heavy objects. Without the Hinst, the robot is unable to autonomously adjust the grip force, leading to slip or damage of some objects.

Movie S4. Comparison between VHT and VT; comparison between VHP based on VHT, VHP based on VT and VP based on VT.

We teach robot to execute pick-and-place tasks with tongs using vision-only approach (VT) and combination of vision and Hinst (VHT) respectively. Then, conducting the practicing with three groups: practice combination of vision and Hinst (VHP) based on VHT (VHP + VHT), VHP based on VT (VHP + VT) and vision-only practice (VP) based on VT (VP + VT).

Movie S5. Robotic home assistance (breakfast service task).

We apply the Hinst robot to a real-life scenario, where it prepares and transports foods, pours water into a cup, and delivers a box of medicine to humans.

Movie S6. Robotic home assistance (handing disabled people and desk-cleaning).

We apply the Hinst robot to a real-life scenario, where it pours water into a cup, assists a disabled individuals walking with self-developed electronic skin (*33*), and cleans the desk.

Movie S7. Employing LLMs and VLMs to accomplish task planning.

The Hinst robot utilizing LLMs and VLMs to do task planning and accomplish organization task.
